# Mithramycin Exerts an Anti-Myeloma Effect and Displays Anti-Angiogenic Effects through Up-Regulation of Anti-Angiogenic Factors

**DOI:** 10.1371/journal.pone.0062818

**Published:** 2013-05-07

**Authors:** Eléonore Otjacques, Marilène Binsfeld, Natacha Rocks, Silvia Blacher, Karin Vanderkerken, Agnès Noel, Yves Beguin, Didier Cataldo, Jo Caers

**Affiliations:** 1 Laboratory of Hematology, Groupe Interdisciplinaire de Génoprotéomique Appliquée (GIGA-Research), University of Liège, Liège, Belgium; 2 Laboratory of Tumour and Development Biology, Groupe Interdisciplinaire de Génoprotéomique Appliquée (GIGA-Research), University of Liège, Liège, Belgium; 3 Department of Hematology and Immunology, Myeloma Center Brussels, Vrije Universiteit Brussel, Brussels, Belgium; Universidade Federal do Rio de Janeiro, Brazil

## Abstract

Mithramycin (MTM), a cytotoxic compound, is currently being investigated for its anti-angiogenic activity that seems to be mediated through an inhibition of the transcription factor SP1. In this study we evaluated its anti-myeloma effects in the syngenic 5TGM1 model *in vitro* as well as *in vivo*. *In vitro*, MTM inhibited DNA synthesis of 5TGM1 cells with an IC50 of 400 nM and induced an arrest in cell cycle progression at the G1/S transition point. Western-blot revealed an up-regulation of p53, p21 and p27 and an inhibition of c-Myc, while SP1 remained unaffected. In rat aortic ring assays, a strong anti-angiogenic effect was seen, which could be explained by a decrease of VEGF production and an up-regulation of anti-angiogenic proteins such as IP10 after MTM treatment. The administration of MTM to mice injected with 5TGM1 decreased 5TGM1 cell invasion into bone marrow and myeloma neovascularisation. These data suggest that MTM displays anti-myeloma and anti-angiogenic effects that are not mediated by an inhibition of SP1 but rather through c-Myc inhibition and p53 activation.

## Introduction

Multiple myeloma (MM) is a haematological malignancy affecting terminally differentiated B cells and characterized by bone marrow (BM) infiltration by plasma cells secreting a monoclonal paraprotein [1]. The disease is heterogeneous with a wide range of clinical manifestations and differing outcomes that are driven by its genetic and biological characteristics [2]. These genetic alterations finally contribute to a malignant phenotype with a capacity for self-renewal and a potential for a low rate of proliferation, in contrast to normal plasma cells [3].

In addition to these genetic changes, an evolving crosstalk between myeloma cells and other cell types within the BM microenvironment can also account for MM development. This crosstalk favours the activation of fibroblasts to secrete different growth factors and of endothelial cells to initiate an angiogenic response. Cells from the BM microenvironment also stimulate immune and inflammatory cells and disrupt the balance between osteoblasts and osteoclasts, leading to osteolysis [4]. Angiogenesis in the BM also seems to play an important role in the pathogenesis of MM [5]. Indeed, increased BM microvessel density (MVD) in patients with MM has been correlated with disease progression and poor prognosis [6]. Rajkumar *et al.* have shown a gradual increase of BM angiogenesis along the disease spectrum from monoclonal gammopathy of undetermined significance to smouldering MM, newly diagnosed MM and relapsed MM [7]. However, gene expression studies showed that normal BM plasma cells already express some pro-angiogenic genes and an accumulation of normal plasma cells can already induce a basal level of angiogenesis in the BM [8]. Aberrant expression of pro-angiogenic genes (vascular endothelial growth factor [VEGF], basic fibroblast growth factor [bFGF], and hepatocyte growth factor [HGF]), and down-regulation of anti-angiogenic genes by MM cells further increase the angiogenic stimulus and explains the presence of BM angiogenesis at various degrees in all myeloma patients [5]. The main problem in the development of anti-angiogenic agents is that multiple angiogenic molecules may be produced by tumours, and tumours at different stages of development may depend on different angiogenic factors for their blood supply.

In the current study, we evaluated the anti-myeloma effects of mithramycin (MTM), a natural polycyclic aromatic polyketide produced by *Streptomyces* species [9]. MTM binds preferentially to GC-rich sequences in DNA corresponding to transcription factor SP (specific protein) binding sites, inhibits expression of SP-regulated genes, and has anticancer activity 10–12. Recently, there has been a renewed interest in this compound because of its newly-described anti-angiogenic effects and the use of analogues with distinct anti-cancer effects [12]–[15].

## Materials and Methods

### Cell Culture

The 5TGM1 myeloma cell line was subcloned from a stroma-independent cell line originally established from the parent murine 5T33MM (IgG_2b_κ) myeloma cell line [16], [17], which arose spontaneously in an aged C57BL/KaLwRij mouse [18]. This cell line was kindly provided by G Mundy (Vanderbilt University, Nashville, TN, USA). The 5TGM.1 model closely reflects different aspects of the MM disease seen in humans. For example, it include the growth of myeloma in bone, the dependency of the myeloma on the bone microenvironment for its growth and survival, the production of a paraprotein that reflects myeloma burden and the associated osteolytic processes. The 5TGM1 murine myeloma cell line was cultured in Dulbecco’s Modified Eagle Medium (DMEM) (Invitrogen, Grand Island, NY, USA) supplemented with 10% heat-inactivated fetal bovine serum (FBS; Thermo Scientific, Rockford, IL, USA), 100 U/ml penicillin–streptomycin, and 2 mM L-glutamine (Invitrogen). 5TGM1 cells have been genetically engineered to stably express eGFP (5TGM1-eGFP cells) [19]. For inoculation into mice, cultured cells were harvested and resuspended in serum-free DMEM at a concentration of 2.5 10^5^ cells/200 µl. The BM endothelial cell line STR-4 and STR-10 were kindly provided by Dr M Kobayashi and also cultured in supplemented RPMI-1640 [20].

### Reagents

MTM was purchased from Sigma-Aldrich (St. Louis, MO, USA) and dissolved in DMSO to obtain a stock solution at 500 µM.

### Proliferation Assay

*In vitro* proliferation and viability was assessed using MTT assay according to the manufactureŕs recommendations (Cell Proliferation Kit I, MTT, Roche Diagnostics GmbH, Mannheim, Germany). Cells were cultured for 24 h in DMEM medium (96-well plates) supplemented with different concentrations of MTM or with the vehicle alone used as control. This culture was followed by a incubation with 10 µL MTT labelling reagent for 4 h at 37°C. Then 100 µL of solubilisation solution were added to each well overnight at 37°C and absorbance at 560 nm was read on a Microplate reader.

### Apoptosis Assay

5TGM1 cells were cultured in 6-well plates at a density of 150,000 cells in 200 µl. Cells were incubated during 24 h with MTM (0 nM, 200 nM, 400 nM, 800 nM and 1600 nM) and harvested. Cells were incubated with FITC-annexin V and propidium iodide (Annexin V: FITC Apoptosis Detection Kit I; BD Biosciences, San Jose, CA, USA) according to the manufacturer’s instructions. Apoptosis was assessed by flow cytometry using a FACS Canto II (BD Biosciences). The numbers indicate percentage of cell population in each quadruplet. Lower left: normal cells; lower right: apoptotic cells; upper right: late apoptotic and necrotic cells.

### Cell Cycle Analysis

5TGM1 cells were cultured in 6-well plates at a density of 150,000 cells in standard culture medium. After 24 h of incubation with increasing concentrations of MTM (0 nM, 200 nM, 400 nM), cells were harvested, washed twice with PBS and fixed in ethanol for 30 min at 4°C. After that, cells were stained with propidium iodide (100 µM/ml in PBS, Sigma). Flow cytometry was performed on a FACS Calibur (BD Biosciences) and the percentage of cells in each phase of the cycle was calculated using the Modfit software (Verity Software House, Topsham, ME, USA).

### Western Blot

After 24 h incubation with different concentrations of MTM, cells were harvested and the cell pellets were washed with PBS. Cells were then lysed in a buffer containing SDS 1%, a protease inhibitor (Thermo Scientific) and a phosphatase inhibitor (phosSTOP; Roche, Mannheim, Germany). Proteins were separated on a 12% acrylamide gel under reducing conditions and transferred to PVDF membranes (Bio-Rad, Hercules, CA, USA). Following the transfer, membranes were blocked for 2 h with PBS containing 5% non-fat dried milk and 0.1% Tween 20 and incubated overnight at 4°C with the appropriate primary antibody namely c-Myc (9E10; Santa Cruz Biotechnology, Santa Cruz, CA, USA), SP1 (AnaSpec, Fremont, CA, USA), cyclin D1 (C-20; Santa Cruz Biotechnology), p21 (C19; Santa Cruz Biotechnology), p27 (C19: Santa Cruz Biotechnology), p53 (Millipore), cyclin B1 (GNS1; Santa Cruz Biotechnology), cyclin E (M-20; Santa Cruz Biotechnology), cdk2 (M2; Santa Cruz Biotechnology), cdk4 (C-22; Santa Cruz Biotechnology), cdk6 (C-21; Santa Cruz Biotechnology), phospho Rb (Ser 780) (9307; Cell Signalling, Danvers, MA, USA). Membranes were washed in PBS-Tween buffer and incubated with the appropriate HRP-conjugated secondary antibody diluted 1∶2000 in PBS containing 5% milk and 0.1% Tween-20. After three washes, bands were visualised using the ECL plus system (Perkin Elmer). B-actin (Sigma-Aldrich) was used as a loading control.

### Endothelial Cell Migration and Wound Healing Assay

For studying chemotaxis of STR-4 and STR-10 cells, uncoated 8-µm Nucleopore filters (Whatman, Middlesex, UK) were used in 48-well invasion chamber (Neuroprobe), as previously described [21]. Cells were treated with 200 nM or 400 nM of MTM or vehicle. Chambers were incubated at 37°C for 24 h and cell were able to migrate. Migration was quantified by counting the number of cells on the lower surface of the filters (30 random fields). Results are expressed as mean number of migrating or invading cells per 30 fields ± SE and are those of one representative experiment done at least three time (each experimental condition done in quadruplicates).

In the wound healing assay, STR-4 and STR-10 cells were seeded at a density of 3.5×10^4^/mL and 4.1×10^5^/mL, respectively, into sterile cell culture inserts (Ibidi GmBH, Munich, Germany). Twenty-four hours after seeding, the cell culture insert was carefully removed, revealing a defined 500-µm gap in the attached cells. Cells were incubated with RPMI medium containng 10% FBS and 200 nM and 400 nM Mithramycin. Endothelial cells were monitored for migration into the wound using a phase contrast microscopy (Zeiss Axiovert 25 microscope equipped with an Axiocam Zeiss camera). Areas without cell were quantified using the ImageJ program.

### *In vitro* Angiogenesis: the Rat Aortic Ring Assay

Before the experiment, conditioned medium (3 ml per condition) was prepared by culturing 10^5^ 5TGM1 cells during 48 h in DMEM supplemented with the vehicle solution or 200 nM or 400 nM of MTM. DMEM was mixed (1∶1) with MCDB, a classical medium used in aortic ring assays. DMEM+MCDB and VEGF were used as negative and positive controls respectively. The rat aortic ring assays were performed as previously described [22]. A computarized method of quantification was used to determine the mean microvessel distribution defined as the number of vessels at various distances from the aortic ring. For this, image processing was performed with the Aphelion 3.2 (Adcis) software and measurements with Matlab (7.9.0) software (Mathworks, Natick, MA, USA), according the algorithm described in Blacher *et al.* [23].

### *In vitro* Angiogenesis: Spheroid Sprouting Assay

Multicellular spheroids were generated by seeding 15000 STR-4 or STR10 cells in each well of non-adherent round-bottomed 96-well plates, in DMEM (Gibco BRL) containing 5% FBS and 0.24% high-viscosity methylcellulose (Sigma-Aldrich) [24]. After 24 hours of culture, spheroids were collected (maximum of eight per well), embedded in collagen gels in 48-well plates, and maintained in 2% FBS DMEM at 37°C for 24 hours. Mithramycin was added at a final concentration of 200 nM or 400 nM. Cells were examined under a Zeiss Axiovert 25 microscope equipped with an Axiocam Zeiss camera and KS 400 Kontron image analysis software (Carl Zeiss Microscopy, Zaventem, Belgium). Images were quantified as earlier described [24].

### *In vivo* Treatment with MTM on the 5TGM1 Mouse Model

C57BL/KaLwRij mice were purchased from Harlan (Horst, Netherlands). Animals were housed under conventional conditions and had free access to food and tap water. All procedures involving these mice were approved by the local animal ethics committee at the University of Liège. For the in* vivo* experiment, C57BL/KaLwRij mice (n = 20) were injected intravenously with 2.5 10^4^ GFP-transfected 5TGM1 cells. Starting the day after injection, one group of mice was treated intraperitonealy twice weekly with MTM (0.750 mg/kg) while the second group was treated with the vehicle (DMSO dissolved in PBS). When mice showed first signs of paraplegia (at day 32), all animals were sacrificed. BM was flushed from one tibia and one femur and BM mononuclear cells were isolated by adding Red Blood cell lysis buffer on the cell suspension (Sigma-Aldrich). Tumour load was determined by flow cytometric analysis of GFP cells. The second tibia and the second femur were fixed for immunohistochemical staining. The bones were fixed by immersion for 48 h in zinc fixative and immersed in a decalcification solution for 48 h. Spleen weight was measured. CD31 staining was performed using immunohistochemistry as described previously [25].

### ELISA for VEGF and IP10

VEGF and interferon gamma-induced protein 10 (IP10) (R&D Systems, USA) were measuredby ELISA on conditioned medium from untreated cells and cells treated with 200 nM or 400 nM of MTM for 24 h according to the manufacturer’s protocol. IP10 ELISA was also performed on lysates of treated and untreated cells.

### Angiogenesis Cytokine Array

Proteome Profiler Mouse Angiogenesis array (R&D Systems) was performed on conditioned medium from untreated cells and cells treated with 400 nM of MTM for 24 h according to the manufacturer’s protocol.

### Statistical Analysis

All experiments were performed in at least triplicates. Results were expressed as score means ± standard error of means (SEM). Statistical analyses were performed by Student t-test using the Instat software (GraphPad, La Jolla, CA, USA) (*p<0.05; **p<0.01; ***p<0.001).

## Results

### *In vitro* Effects of MTM on 5TGM.1 Cell Proliferation and Cell Cycle Progression

**Figure 1:**
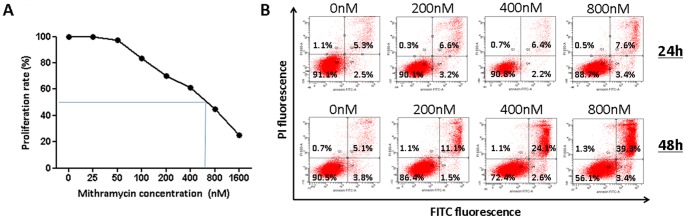
MTM inhibits proliferation and induces apoptosis in 5TGM1 cells. **A** Effect of MTM on 5TGM1 cell proliferation and viability. Cells were treated with various concentrations of MTM for 24 h and viability was assessed using an MTT assay. There was a dose dependent inhibition in DNA synthesis observed with an IC50 of 600 nM **B** The induction of apoptosis was determined by FACS analysis. Cells were incubated during 24 h or 48 h with MTM (0 nM, 200 nM, 400 nM and 800 nM) and annexin V/PI stainings were performed. A representative picture of FACS analysis shows no induction of apoptosis after 24 h and the presence of late apoptotic cells after 48 h of treatment.

We initially assessed the effect of MTM on 5TGM1 cell proliferation and viability *in vitro* using aMTT assay. In this setting, 5TGM1 cells were treated for 24 h with increasing concentrations of MTM (ranging from 25 nM to 800 nM). As shown in **Figure 1** **A**, treatment with MTM induced a dose-dependent decrease of cell proliferation and the median inhibitory concentration (IC50) was 600 nM. To determine whether the reduction in 5TGM1 cell proliferation was accompanied by apoptosis induction, cells were incubated with MTM or vehicle for 24 h and 48 h and early and late apoptosis rates were assessed by flow cytometry using a double annexin V/PI-staining (**Figure 1** **B**). When cells were treated for 24 h with the drug, no effect on early apoptosis was observed. However, a significant effect on late apoptosis and necrosis was observed with high concentrations of MTM (p<0.01). After 48 h of MTM treatment, a dose-dependent increase of late apoptosis/necrosis rate was observed in each condition compared to the control condition.

**Figure 2:**
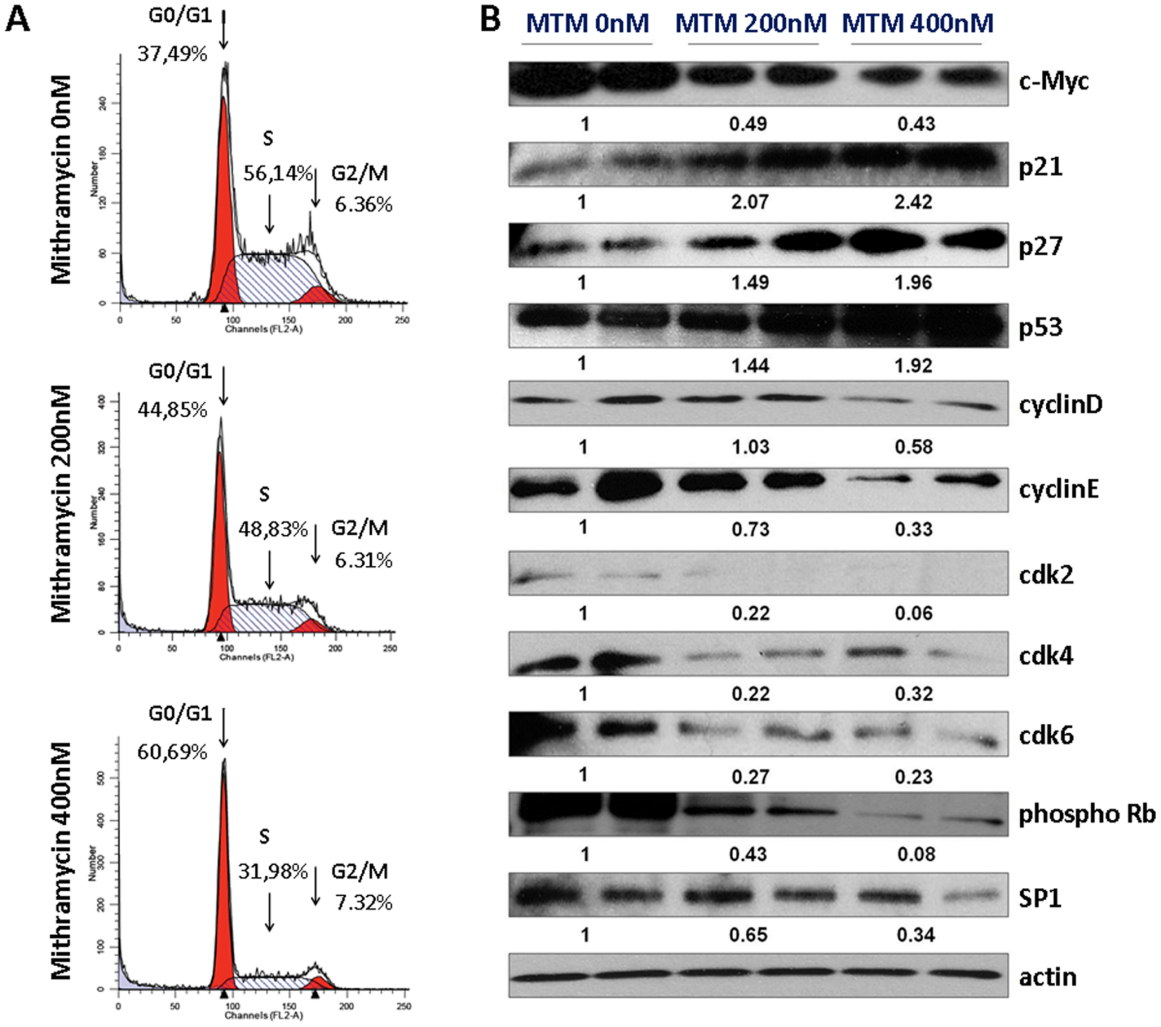
MTM induces a dose-dependent increase of the percentage of cells in G0/G1 phase. **A** Cell cycle analysis of 5TGM1 cells treated with 0, 200 or 400 nM of MTM. Cells were treated for 24 h with 200 nM and 400 nM of MTM and DNA was stained with propidium iodide, followed by flow cytometry analysis. This treatment resulted in a dose-dependent increase of the percentage of cells in G0/G1 phase (45% and 61%, respectively) when compared to the untreated cells (37%) (p<0,01). A decreased percentage of cells in the S phase was also observed (56% versus 49% and 32% for the 200 nM and 400 nM conditions, respectively) (p<0,01). Percentages of cells in G1, S or G2 phases following MTM treatment are indicated on the graph. **B** Protein expression of c-Myc, p21, p27, p53, cyclin D/CDK4 and cyclin E/CDK2, cdk6, phospho Rb and SP1 analyzed by Western blot (duplicates, repeated 3 times). Protein loadings were normalized with β-actin. Data shown are representative of at least three independent experiments. We observed an upregulation of p53, an inhibition of c-myc, while SP-1 remained stable. Downstream, p21 and p27 were upregulated, while cyclin B, cyclin E, CDK2, CDK4, CDK6 were decreased, which finally resulted in decreased phosphorlyation of Rb.

To determine whether MTM affects cell cycle progression in 5TGM1 cells, cells were treated for 24 h with 200 nM and 400 nM of MTM and the DNA was stained with propidium iodide, followed by flow cytometry analysis. As shown in **Figure 2** **A**, treatment of cells with 200 nM and 400 nM of MTM resulted in a dose-dependent increase of the percentage of cells in G0/G1 phase (45% and 61%, respectively) when compared to the non-treated cells (37%) (p<0,01). A decreased percentage of cell in the S phase was also observed (56. % versus 49% and 32% for the 200 nM or 400 nM conditions, respectively) (p<0,01). Knowing that MTM acts through inhibiting SP1 or c-Myc, we investigated protein expression of these two transcription factors by Western blot. These experiments showed a reduction of c-Myc protein levels, while SP1 levels were unaffected (**Figure 2** **B**). Since p53 signalling cascade is known to play a role in the cell cycle arrest in response to cellular stress like DNA damage [26], p53 production was investigated using Western blot. Following MTM treatment, a distinct increase in p53 protein expression was observed (**Figure 2** **B**). Moreover, to understand the molecular mechanisms underlying the observed MTM-induced inhibition of cell growth [27], the expression of several intracellular regulators of the cell cycle was assessed by Western blot analysis (**Figure 2** **B**). For this, 5TGM1 cells were treated for 24 h with 200 nM and 400 nM of MTM. MTM exposure resulted in a significant reduction of the expression of cell cycle regulatory proteins, cyclins D1, E, CDK2, CDK4, CDK6 and c-Myc, particularly with 400 nM treatment. Knowing that CDK4, CDK6 and also Cyclin E, through activation of CDK2, are responsible for phosphorylation of pRb, the effect of MTM on the expression of pRb was investigated. MTM treatment caused a decrease of pRb phosphorylation in 5TGM1 cells. Because p21 and p27 mediate the p53-dependent G1 phase arrest in response to a variety of stress stimuli, their protein expression was evaluated by Western blot, showing an increase in their protein expression (**Figure 2** **B**). In summary, cell cycle arrest at the transition of G1 to S phase seems mediated by a decreased expression of cell cycle inducers (such as cyclins and cyclin-dependent kinases) but also by an increased expression of inhibitors such as p21 and p27 (which are under control of p53).

### Anti-angiogenic Effects of MTM in the Rat Aortic Ring Assay and Secretion of Angiogenic Factors

**Figure 3:**
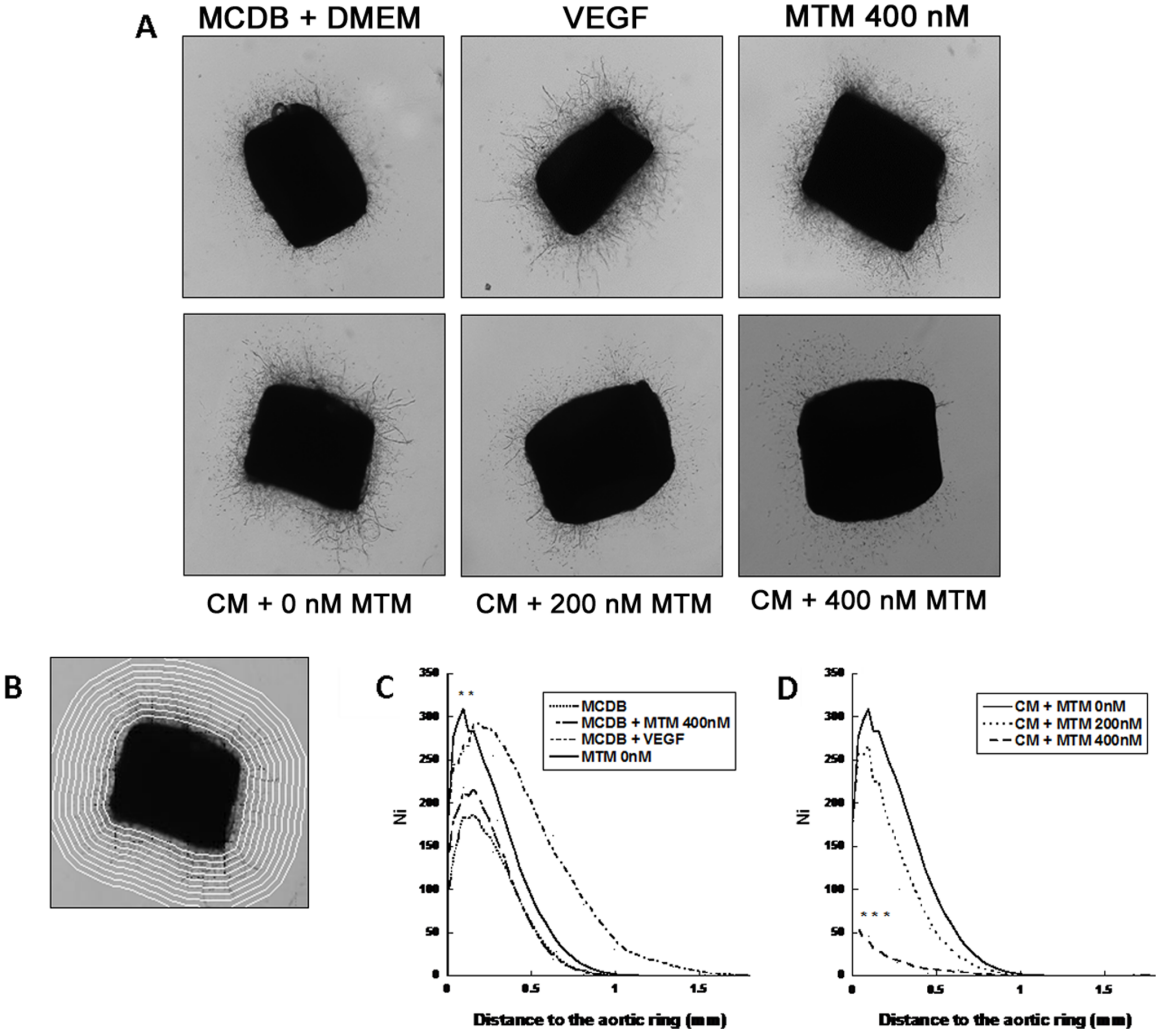
MTM reduced the myeloma-associated neo-vascularization *in vitro* in a rat aortic ring assay. **A** Representative photomicrographs (scale 25×) of aortic rings cultured for 9 days with MCDB+DMEM, VEGF, MTM 400 nM, or with conditioned medium (CM) by cancer cells treated with MTM 0 nM, 200 nM or 400 nM. **B** A grid of concentric rings is automatically drawn by making successive dilations of the aortic ring boundary. Microvessel distribution is defined as the number of microvessel intersections with this grid plotted as a function of the distance from the ring. **C** and **D** represent the mean numbers of vessels depending of the distance to the ring for control (**C**) and MTM treatment (**D**). The addition of medium conditioned by treated 5TGM1 cells to the aortic ring culture increased the development of microvessels compared to the control condition (MCDB+DMEM) (p<0.01). This angiogenic response was inhibited (p<0.001) when increased concentrations of MTM (200 nM and 400 nM) were added to 5TGM1 cells before collection of conditioned medium.

Angiogenesis was first assessed *in vitro* by using the rat aortic ring assay (**Figure 3** **A**). **Figure 3** **C** and **3 D** show the mean number of vessels in function of the distance to the ring. The addition of medium conditioned by untreated 5TGM1 cells to the aortic ring culture increased the development of microvessels compared to the control condition (MCDB+DMEM) (p<0.01) (**Figure 3** **C)**. Direct addition of MTM in the rat aortic ring assay showed no effect on vascular outgrowth (**Figure 3** **C)**, indicating that the observed effect was mediated through an effect on 5TGM1 cells. When increasing concentrations of MTM were added (200 nM or 400 nM) to 5TGM1 cells before collection of conditioned medium, this angiogenic response was inhibited (p<0.001) (**Figure 3** **D**).

**Figure 4:**
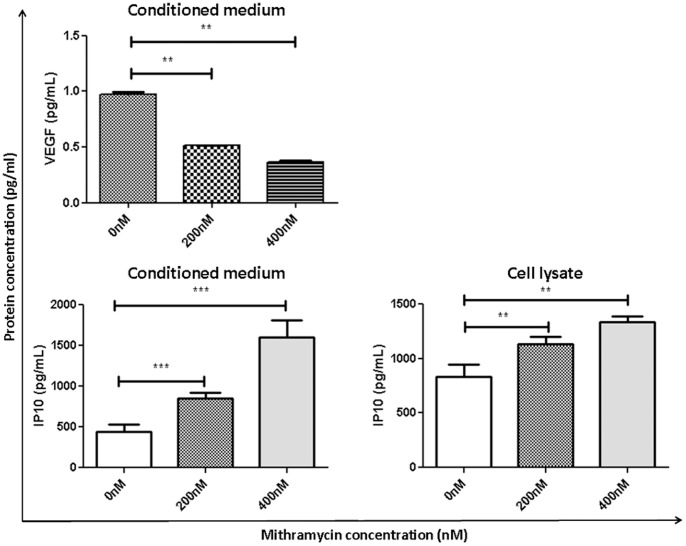
MTM induces decreased VEGF and IP10 secretion. Levels of VEGF were determined in medium conditioned by 5TGM1 cell following 24 h of MTM treatment. Levels of IP10 were determined in both conditioned medium and cell lysate. Results were obtained using ELISA assays.

As VEGF plays a crucial role in angiogenesis, the expression of this protein was assessed by ELISA in the conditioned medium of cultured myeloma cells (**Figure 4**). After MTM treatment (200 nM and 400 nM), VEGF production was significantly (p<0.01) decreased. To assess the expression of other cytokines and to obtain a more global view of the effect of MTM on angiogenesis, a cytokine array was performed on the conditioned medium of treated (400 nM) and the non-treated 5TGM.1 cells (**Figure S1 A** and **B**). A down-regulation of several pro-angiogenic factors as well as an up-regulation of anti-angiogenic factors was found following MTM treatment. Notably, among the tested cytokines, serpin E1 and IP10 were both found to be up-regulated in the treated conditioned medium (**Figure S1 C**). In particular, the member of CXC sub-family IP10 is known to be expressed by endothelial cells and to be a potent angiogenesis inhibitor *in vivo* [28]. Considering its acknowledged role in angiogenesis, IP10 expression was also evaluated by ELISA in both cell lysates and conditioned medium (**Figure 4**). Following MTM treatment (200 nM and 400 nM, respectively), IP10 secretion was significantly induced both in 5TGM.1 cell lysates (p<0.01 for both concentrations) and conditioned medium (p<0.01 for 200 nM; p<0.001 for 400 nM).

### MTM Shows Distinct Anti-tumor and Anti-angiogenesis Effects *in vivo*

**Figure 5:**
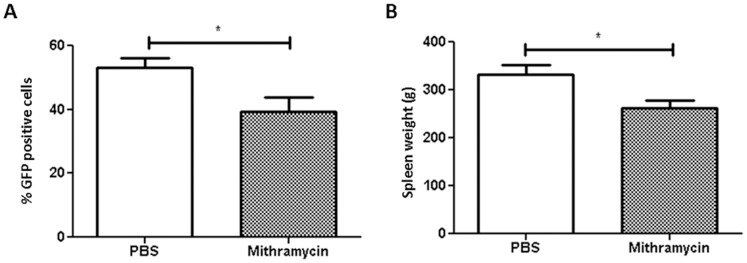
MTM reduced BM colonisation by 5TGM1 (A) and splenomegaly (B). Tumour load was assessed by flow cytometry analysis of GFP cells and spleen weight was measured. Starting the day after injection (day 1), a group of mice was treated intraperitonealy twice weekly with MTM or vehicle (DMSO dissolved in PBS). At day 32, mice were sacrificed.

Intraperitoneal treatment with MTM was well tolerated and did not induce any weight loss (results not shown). The treatment of mice with MTM decreased BM infiltration by 5TGM1 cells with 25% compared to vehicle treated 5TGM1 mice (p<0.05) (**Figure 5** **A**). Splenomegaly was also significantly reduced by 21% after chronic treatment of animals with MTM compared to treatment with vehicle (p<0.05) (**Figure 5** **B**).

**Figure 6:**
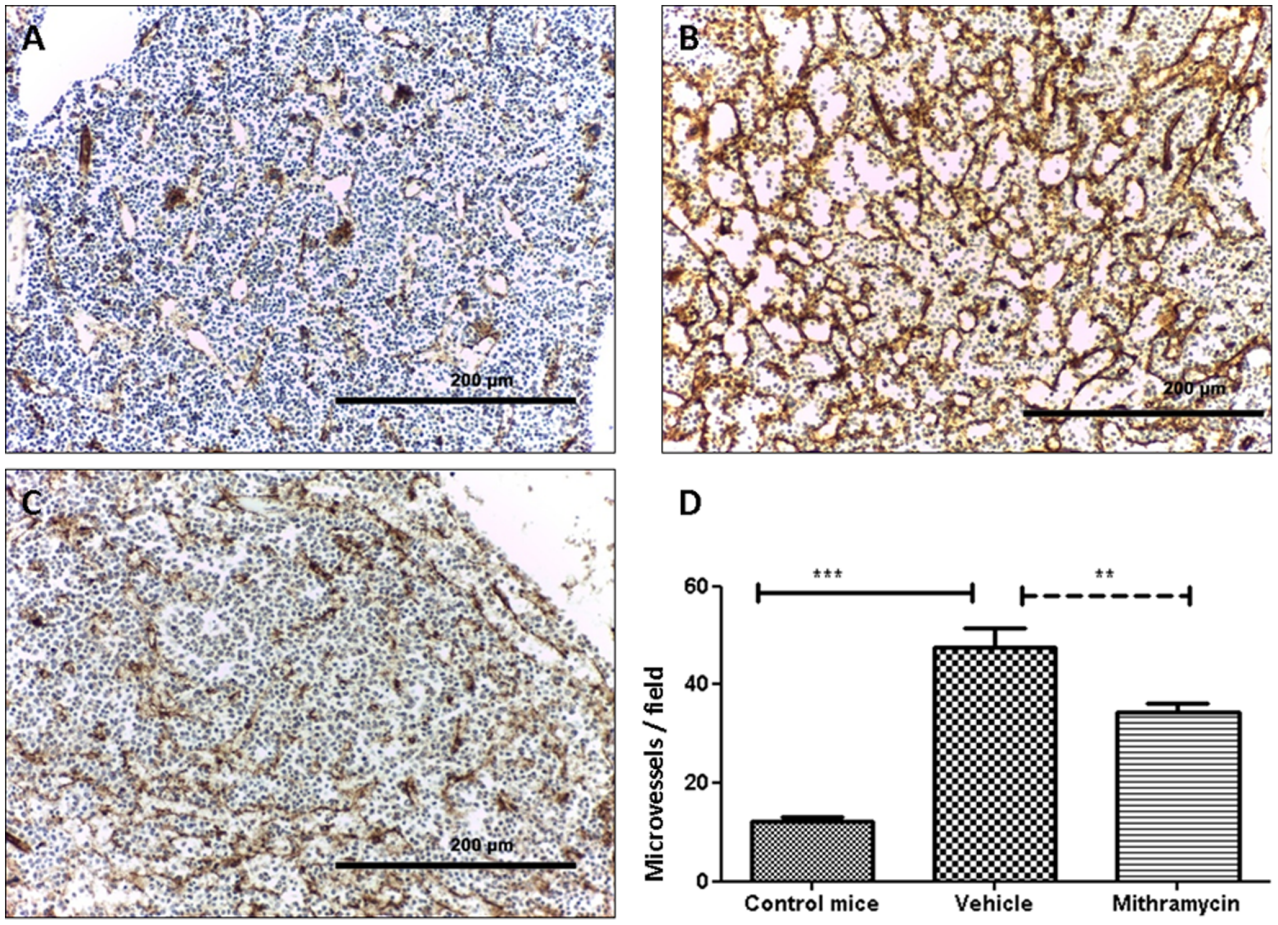
MTM decreased angiogenic response. CD31 immuno-staining on bone marrow sections of control mice (**A**) or 5TGM1 injected mice, treated with the vehicle solution (**B**) or with MTM (**C**). Original magnification 10x. **D** Quantification of microvessel density (MVD) using the hot spot technique. MVD was decreased in MTM-treated mice compared with the group treated with the vehicle solution.

Immunohistochemical staining of CD31 on BM sections of 5TGM1-inoculated mice and age- and sex-matched control mice, demonstrated an increase of the microvessel development in the 5TGM1 injected mice (p<0.001) (**Figure 6**). MTM treatment resulted in a decreased angiogenic response (p<0.01). Angiogenesis quantification is shown in **Figure 6** **D**.

### Direct Effect of MTM on BM Endothelial Cells

**Figure 7:**
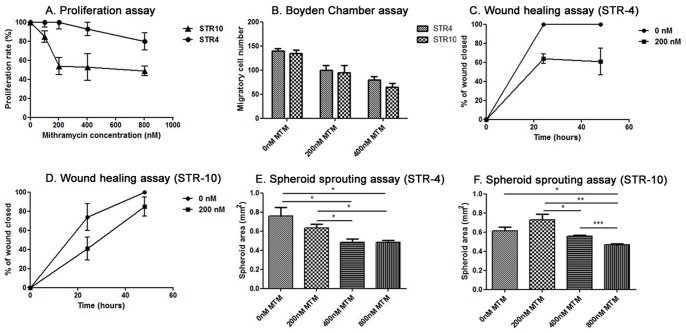
MTM inhibits proliferation, migration and invasion of endothelial cell lines STR4 and STR10. Proliferation and viability was assessed using an MTT assay. Wound healing assay and Boyden chamber assay were used to study migration. Cell sprouting was assessed using a spheroid sprouting assay. Cells were treated with various concentrations of MTM during 24 h and 48 h. (**A**) Increasing concentrations of MTM resulted in a minor anti-proliferative effect in STR-4 cells, while MTM inhibited STR-10 proliferation with an IC-50 of 200 nM. (**B**) MTM inhibited both STR-4 and STR-10 cell migration in the Boyden Chamber assay. (**C,D**) Wound healing assay showed that adding 200 nM MTM decreased cell repopulation by both cells lines. In the spheroid sprouting assay (**E, F**), treatment with 200 nM or 400 nM had only an effect on the STR4 cell line whereas 800 nM of MTM caused an significant decrease in vessel sprouting by both cell lines.

To assess the direct effect of MTM on murine bone marrow endothelial cells, we used the immortalized BM endothelial cell lines STR-4 and STR-10. In the MTT assay, increasing concentrations of MTM (ranging from 100 nM to 800 nM) resulted in a minor anti-proliferative effect in STR-4 cells, while MTM inhibited STR-10 proliferation with an IC-50 of 200 nM (shown in **Figure 7** **A**). MTM inhibited both STR-4 and STR-10 cell migration in the Boyden Chamber assay. As shown in **Figure 7** **B** and **Figure S2 C**, treatment with 200 nM and 400 nM reduced migration with 1,25-fold (200 nM STR4), 1,74-fold (400 nM STR4), 1,41-fold (200 nM STR10) and 1,66-fold (400 nM STR10) compared to vehicle treated cells. To confirm the anti-migratory effects of MTM, a wound healing assay using a IBIDI culture insert was performed. As shown in **Figure 7** **C and 7 D**, treatment with MTM induced a decrease of cell migration in both cell lines (61% and 85% of wound closed after 48 h of treatment with 200 nM of MTM for STR-4 and STR-10 respectively compared to total closure of control condition). Results of this assay are also illustrated in **Figure S2 A** Capillary-like sprout formation from spheroid cultures was only inhibited at high concentrations (800 nM) of MTM in both STR-4 and STR-10 endothelial cells. Lower concentrations of MTM (200 and 400 nM had only significant effect on STR-4 (**Figure 7D** and **Figure S2** **B**), while sprouting by STR-10 cells was unaffected at these concentrations (**Figure 7F**).

## Discussion

In the current study, we have evaluated the *in vitro* and *in vivo* anti-myeloma effects of the known cytotoxic agent MTM. There has been a renewed interest in this molecule since interactions of this molecule with the SP1 pathway have been reported and because of a newly described anti-angiogenic effect [10]–[12]. SP1 has earlier been described to modulate autocrine interleukin (IL)-6 secretion by MM cell lines, affecting their growth [29]. This secretion is dependent on upstream activation of ERK (extracellular signal-regulated mitogen-activated protein kinase), finally resulting in SP1-induced IL-6 transcriptional activation, in turn promoting cell growth. Addition of MTM results in decreased IL-6 production and myeloma cell growth, whereas the ERK inhibitor U0126 strongly inhibits SP1 recruitment at the IL-6 promoter site [29]. More recently, SP1 was described as an important transcription factor in MM cells [30]. Indeed, the authors described a high nuclear expression and activity of SP1, associated with increased DNA binding and SP1-responsive promoter activity. Addition of siRNA and shRNA for SP1 inhibited MM cell growth and survival, while terameprocol, a molecule that competes with SP1-DNA binding, inhibited *in vitro* and *in vivo* MM growth [30].

In our MM model, MTM showed *in vitro* a direct anti-proliferative effect with an IC50 of 600 nM, which is in range with previous reports. MTM inhibited cell proliferation and induced a distinct G0/S1 cell cycle arrest. In addition to the anti-proliferative effects observed, MTM also induced cell death at high concentration. Whereas proliferation and cell cycle progression were inhibited after 24 h, the effects of MTM on apoptosis were only seen at a later time point (48 h) with cells in late apoptotic/necrotic range.

Further investigations on proteins implicated in the control of cell cycle progression from G1 to S phase showed a decrease of protein expression of cyclin D1, cyclin D2, CDK2, CDK4 and CDK6. Considering the fact that up-regulation of cyclins [3] and phosphorylation of CDKs [31] are implicated in cell cycle progression in MM, the observed decreased protein expression of these proteins following MTM treatment can explain the cell cycle arrest. Western blot did not show any decrease in SP1 protein level after MTM treatment. These results indicate that either SP1 is not implicated in mediating the direct effects of MTM or that MTM acts by inhibiting the binding of the SP1 transcription factor to the promoter site of different genes. In this latter case however, one would expect a decreased protein level, because SP1 can bind its own promoter and induce an autonomous loop [32]. One of the downstream genes regulated by SP1 is c-Myc that also controls transition from G1 to S phase during cell cycle progression [11]. In contrast to SP1, we observed a dose-dependent decrease in c-Myc protein production. These results raise some doubts on the initial results of Gerlo *et al.* showing a close relation between SP1 and IL-6 secretion in MM cells by using MTM as a selective SP1 inhibitor [29]. The decreased expression of IL-6 that was observed after MTM treatment may be due to other pathways.

Earlier studies indicated that MTM is a strong activator of endogenous p53 protein in human hepatoma cells [33]. Our results confirm that p53 protein expression was up-regulated following MTM treatment. These results are in line with earlier results in gynaecologic cancer cell lines, where MTM increased the p53 activity through the initial suppression of mdm2 promoter activity, which resulted in increased expression levels of p53 downstream target genes (e.g. PUMA and p21), and induced G0/G1 arrest and apoptosis [34].

In our model, a clear increase was seen in protein levels of p21^Cip1^ indicating that the p53 pathway remained active and that there was no inhibition of transcriptional activation of p21^Cip1^ promoter mediated by SP1. Taken together, our results suggest that the anti-proliferative effects of MTM are mediated by an inhibition of c-Myc and an activation of p53 resulting in decreased expression of cyclins and cyclin-dependent kinases and over-expression of cell-cycle regulatory proteins p27^Kip1^ and p21^Cip1^.

In addition to its direct effect on malignant cells, MTM may inhibit angiogenesis in several ways. First, it can bind to the GC boxes in the promoter sequences of key genes that regulate angiogenesis and inhibits SP1-stimulated transcription of genes such as VEGF/VEGFR, bFGF/bFGFR, EGF/EGFR, IGF/IGFR, PDGF and others. Further, it has the ability to inhibit the binding of SP1 to GC boxes in promoter sequence of SP1 itself, thereby inhibiting the autocrine amplification loop for SP1 signalling. In xenograft models of human cancer, the group of Keping Xie *et al.* demonstrated single agent anti-tumour activity in a variety of xenograft models (carcinoid, gastric cancer and pancreatic cancer cell lines) [35], [36]. In addition, additive or synergistic activity was demonstrated when combined with agents targeting the VEGF pathway. The authors showed that treatment with Bevacizumab, a monoclonal antibody that inhibits vascular endothelial growth factor, increased the expression of SP1 and its downstream molecule VEGF, which could be suppressed by MTM [35]. Therefore, the authors suggested that neutralization of VEGF function by Bevacizumab may up-regulate the expression of SP1 via a positive feedback loop. Resistance to this treatment with increased VEGF expression could be overcome by MTM.

Herein, for the first time, we observed that MTM treatment reduced angiogenesis both *in vitro* and *in vivo* in multiple myeloma. We noticed that the decrease of angiogenesis was not the result of exaggerated cell death or of a global down-regulation of pro-angiogenic factors but was the consequence of the up-regulation of anti-angiogenic factors. In this study, we raise different arguments in favour of such an up-regulation of anti-angiogenic genes. First, direct addition of MTM to rat aortic ring assays had no effect on vascular outgrowth. Second, the effect was only seen when MTM was added during incubation of 5TGM1 for preparing conditioned medium. Third after 24 h there was no increase in apoptosis or cell death as seen with flow cytometry for annexin V/PIA or trypan blue staining. A final proof of this concept was afforded by the cytokine array that showed a global down-regulation of pro-angiogeneic proteins but up-regulation of anti-angiogenic genes. Among them, IP10, a member of the CXC sub-family known to be an inhibitor of angiogenesis *in vivo*, was found to be highly up-regulated. Interestingly, the IP-10 promoter does not contain a GC box and its expression does not seem to be mediated by SP1 [37]. The 5-flanking promoter region of IP10 contains multiple regulatory elements, including two interferon-stimulated responsive elements, two signal transducers and activators of transcription, two CCAAT/enhancer-binding proteins β (C/EBP-β), and two nuclear factor κB (NF-κB)-binding sites [38]. Complementary studies on the BM endothelial cell lines STR-4 and STR-10 showed a possible direct effect of MTM on endothelial cells. Indeed, MTM showed distinct effects on endothelial cell migration, while proliferation was less affected. These results are in line with an earlier observation showing that similar doses of MTM had no significant effect on apoptosis of HUVEC cells but affected DNA synthesis (37% decrease for 300 nM MTM) [39]. Inhibition of proliferation and migration resulted in decreased repopulation in the wound healing assay, but significant effects in a spheroid sprouting assay could only be seen at very high doses of MTM. Anti-angiogenic effects of other chemotherapeutic agents have been described earlier [40], [41]. Hence, we previously described an anti-myeloma and anti-angiogenic activity of Plitidepsin, a cyclodepsipeptide originally isolated from the Mediterranean tunicate *Aplidium albicans*, that is currently under investigation in a phase III study in MM. [42] The anti-angiogenic effect observed of MTM in this study is based on an upregulation of anti-angiogenic factors (such as IP-10) and a most likely effect on proliferation and migration of endothelial cells.

Chronic intraperitoneal treatment with MTM also resulted in a significant decrease of 5TGM1 cell invasion in the mice BM. As myeloma cells accumulate into the spleen in this model, splenomegaly was also reduced following MTM treatment. We believe that the current results sustain the idea that neovascularisation is induced by the presence of myeloma cells and that the observed decrease in angiogenesis probably contributes to the anti-myeloma effects but this may not the sole explication. Indeed, treatment of a xenograft myeloma model with the kinase inhibitor dasatinib reduced myeloma-induced angiogenesis to basal levels, but there was still tumour progression [43]. This indicates that neo-vascularization is not required for final tumor development. The observed effects in our model are thus probably due to both anti-myeloma effects and anti-angiogenic effects. In spite of these encouraging results, the use of MTM is limited due to its toxic side effects and its small therapeutic range [44]. The introduction of new analogues and other drug preparations, such as liposomal formulations and its recent anti-angiogenic effects renew the current interest in this molecule [14], [45]–[47].

In conclusion, MTM treatment resulted in a clear anti-myeloma activity, both *in vitro* and *in vivo*. MTM treatment resulted in a significant effect against various endpoints of the multiple myeloma disease, such as BM invasion, malignant cell proliferation, cell cycle progression and angiogenesis. Importantly, we have highlighted that anti-angiogenic effects of the drug result from an up-regulation of anti-angiogenic factors. Despite the toxic range limitation of MTM, our results suggest that the use of MTM drug analogs or liposomal formulations, or combination with other therapeutic agents are worth considering for further preclinical studies in MM disease.

## Supporting Information

Figure S1 **Proteome profiler mouse angiogenesis array.** Cytokine array was performed on conditioned medium of 24 h non treated cell and treated cell with 400 nM of MTM. **A** Representative cytokine array. The block arrows represent the serpin E1 expression and the dotted arrow the IP10 expression. **B** Diagram represents the mean signal intensity (AU) of the expressed cytokines in treated (400 nM) and non treated (0 nM) conditions. **C** Diagram represents the two major cytokines modulated: serpin E1 and IP10.
(TIF)

Figure S2 **Illustrations of migration assays and sprouting assay.** **A Wound healing assay.** BM endothelial cell line STR-4 was in cultured in an Ibidi cell culture inserts that causes a 500 µm gap in the monoloyer. After incubation with RPMI-1640 with 10% FBS, this gap was completely repopolulated after 24 h by migrating endothelial cells. Treatment with 200 nM MTM abrogated this process resulting in a persistent opening. **B**. **Spheroid sprouting assay** Spheroids consisting of endothelial cells cultured in methylcellulose were moved in collagen gels and endothelial cell out growth and capillary-like sprout formation was followed. Addition of MTM reduced cell outgrowth and sprouting formation compared to the control condition. These differences were significant at 400 nM and 800 nM. **C Boyden Chamber assays**. Representative photomicrographs of migrated STR-4 cells at 20x magnification. Both 200 and 400 nM MTM inhibited endothelial cell migration to the lower chamber.
(TIF)
