# Supplementary figures and images for: Mithramycin Exerts an Anti-Myeloma Effect and Displays Anti-Angiogenic Effects through Up-Regulation of Anti-Angiogenic Factors
Source: PLoS One. 2013 May 7;8(5):e62818. doi: 10.1371/journal.pone.0062818 (PMC3646989; doi:10.1371/journal.pone.0062818)

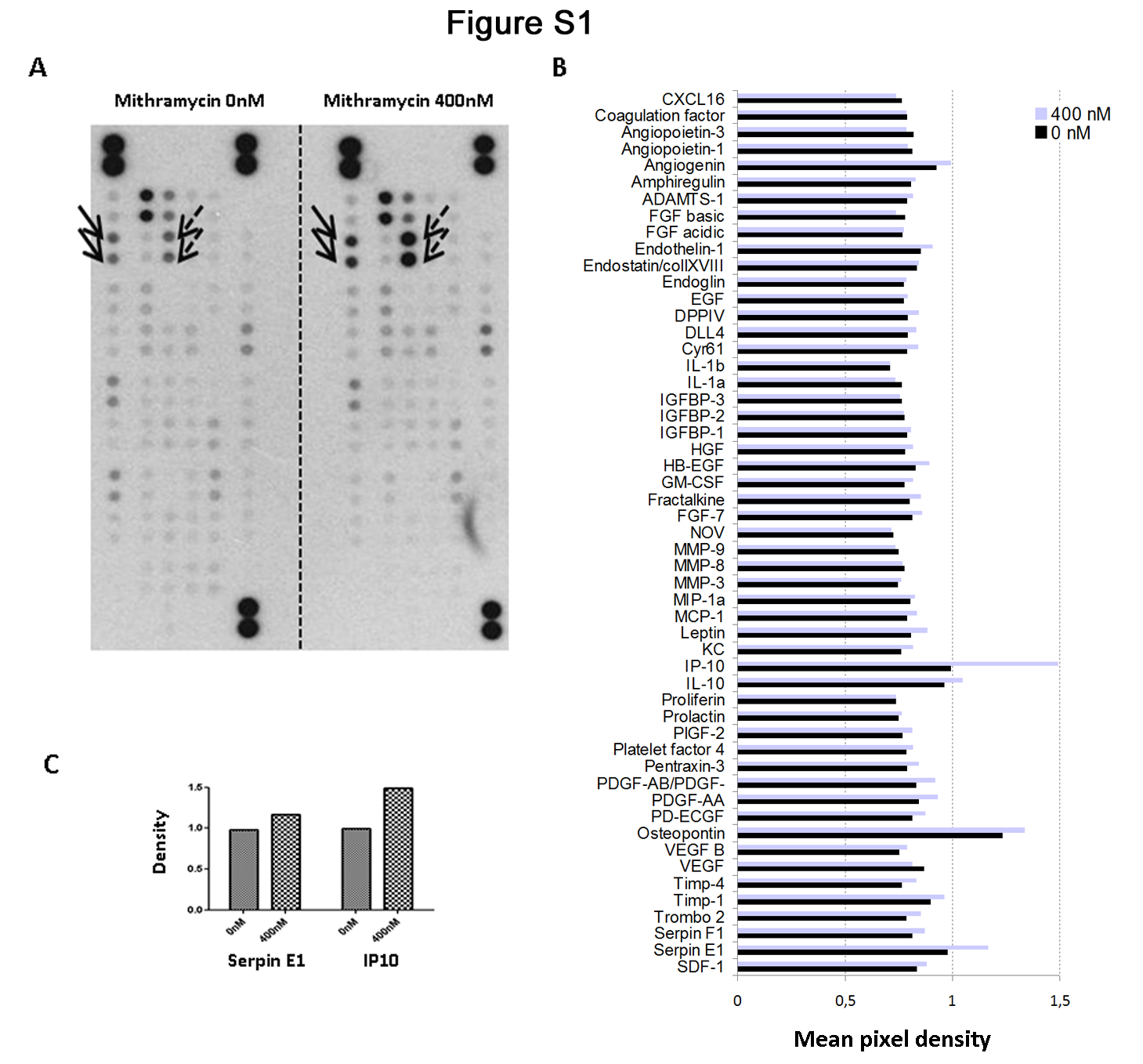

Supplement: Figure S1 — Proteome profiler mouse angiogenesis array. Cytokine array was performed on conditioned medium of 24 h non treated cell and treated cell with 400 nM of MTM. A Representative cytokine array. The block arrows represent the serpin E1 expression and the dotted arrow the IP10 expression. B Diagram represents the mean signal intensity (AU) of the expressed cytokines in treated (400 nM) and non treated (0 nM) conditions. C Diagram represents the two major cytokines modulated: serpin E1 and IP10. (TIF) [file pone.0062818.s001.tif]

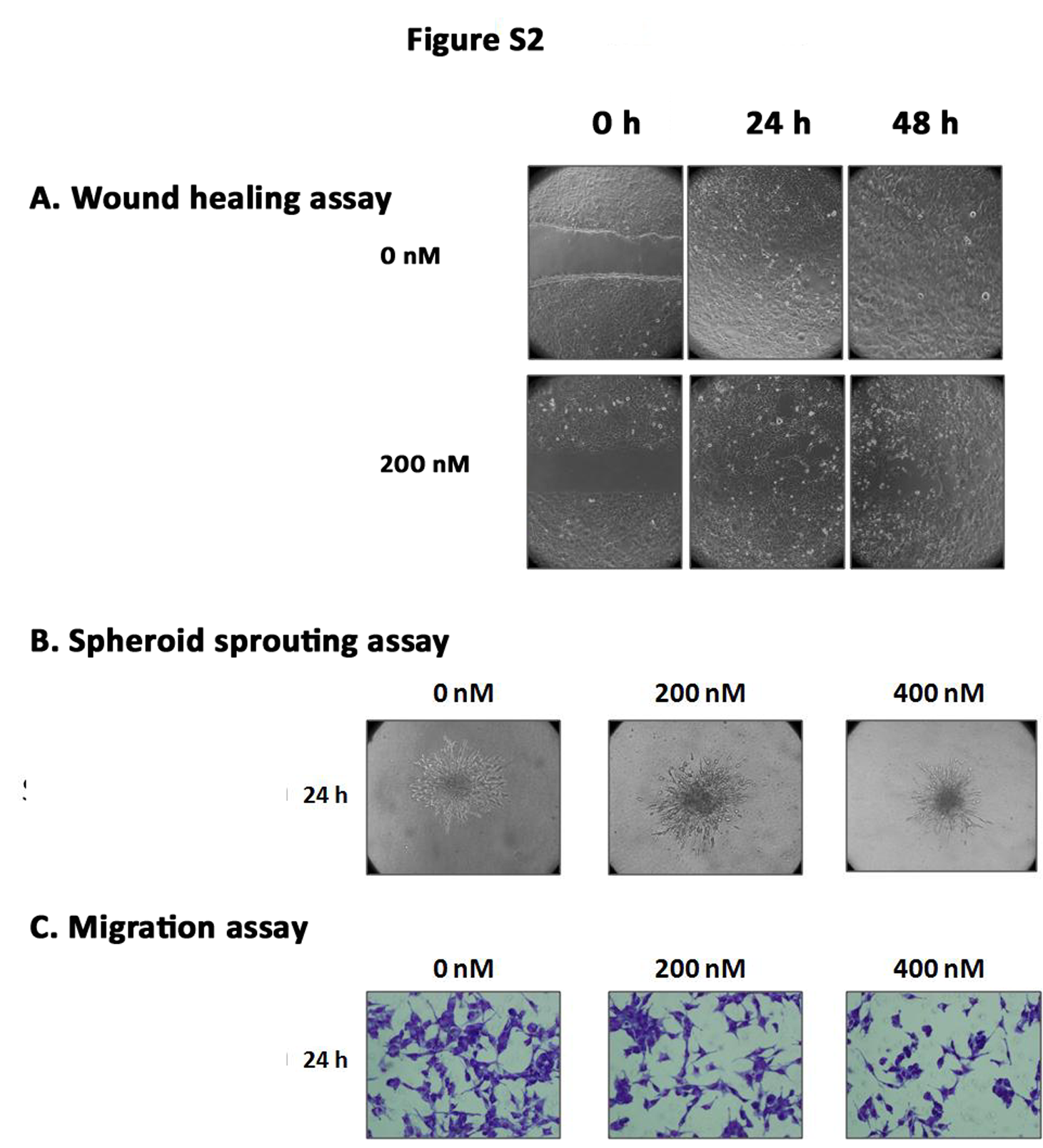

Supplement: Figure S2 — Illustrations of migration assays and sprouting assay. A Wound healing assay. BM endothelial cell line STR-4 was in cultured in an Ibidi cell culture inserts that causes a 500 µm gap in the monoloyer. After incubation with RPMI-1640 with 10% FBS, this gap was completely repopolulated after 24 h by migrating endothelial cells. Treatment with 200 nM MTM abrogated this process resulting in a persistent opening. B. Spheroid sprouting assay Spheroids consisting of endothelial cells cultured in methylcellulose were moved in collagen gels and endothelial cell out growth and capillary-like sprout formation was followed. Addition of MTM reduced cell outgrowth and sprouting formation compared to the control condition. These differences were significant at 400 nM and 800 nM. C Boyden Chamber assays. Representative photomicrographs of migrated STR-4 cells at 20x magnification. Both 200 and 400 nM MTM inhibited endothelial cell migration to the lower chamber. (TIF) [file pone.0062818.s002.tif]
